# Different Effects of Antioxidants Against Ionizing Radiation: An Experimental Model of Micronuclei

**DOI:** 10.3390/cimb48040364

**Published:** 2026-03-31

**Authors:** Miguel Alcaraz, Daniel Gyingiri Achel, José Antonio Garcia-Gamuz, Miguel José Ruiz-Gómez, Ana Mercado-Díaz, Miguel Alcaraz-Saura, José Luis Navarro-Fernández, Amparo Olivares

**Affiliations:** 1 Radiology and Physical Medicine Department, School of Medicine, Campus de Excelencia Internacional de Ámbito Regional (CEIR)-Campus Mare Nostrum (CMN), University of Murcia, 30100 Murcia, Spain; gamuz@um.es (J.A.G.-G.); anamaria.mercado@um.es (A.M.-D.); miguel.alcaraz@um.es (M.A.-S.); jlnf@um.es (J.L.N.-F.); amparo.o.r@um.es (A.O.); 2 Department of Medical Physics, School of Nuclear and Allied Sciences, University of Ghana, Atomic Campus, Legon, Accra GE-292-9709, Ghana; daniel.achel@gaec.gov.gh; 3 Radiology and Physical Medicine Department, School of Medicine, University of Málaga, 29010 Málaga, Spain; mjrg@uma.es

**Keywords:** radiation effects, radioprotectors, micronucleus, polyphenols, diterpenes, rosmarinic acid

## Abstract

There is an urgent need to find and develop radiation countermeasures for both planned and unplanned exposures. However, in recent years, radioprotective substances, such as rosmarinic acid (RA), have been described, presenting contradictory and even paradoxical results. In this paper, we evaluated the genoprotective capacity of 29 substances against chromosomal damage induced by gamma radiation in a comparative study using the same technique, i.e., cytokinesis-block micronucleus (CBMN) assay to evaluate their genoprotective capacities, at the same concentrations and administered before and after exposure to 2 Gy of gamma radiation. We then related the observed effects with their chemical characteristics to appreciate the different mechanisms of action that could explain some apparent contradictions that may emerge. In our study, before exposure to ionizing radiation, RA produced the greatest reduction in the frequency of radiation-induced micronuclei (*p* < 0.001), presenting the highest magnitude of protection (58%) and a dose reduction factor of 7.1 (*p* < 0.001); however, it loses this genoprotective capacity when administered after exposure to radiation. These results could be attributed to the different radical-scavenging and antilipoperoxidative activities of each substance tested. Antilipoperoxidant activity was found to be the most important factor in the reduction in radiation-induced chromosomal damage; thus, lipo-antioxidant substances emerged as the most effective in protecting genetic material against oxidative damage.

## 1. Introduction

There is a clear need for the development of novel substances that can mitigate the undesirable effects of ionizing radiation in a wide array of its medical applications, especially in diagnostic imaging, nuclear medicine, and radiation oncology directed at both patients and professionally exposed workers. Similarly, the risk of accidents and potential terrorist attacks at nuclear facilities that expose populations has increased interest in this aspect of radiological protection [1,2,3,4,5]. In addition, these substances possess the hope of reducing the harmful effects produced by exposure to cosmic radiation, currently considered as one of the limiting factors in space exploration activities [6,7].

Since the discovery of sulfhydryl compounds as radioprotective agents, it has been observed that, for these groups of compounds to be effective, they must be present in the biological environment at the time of radiation; thus, new substances with greater effectiveness and less toxicity have been sought. In recent decades, new substances have been discovered with radioprotective or genoprotective capacities against damage induced by ionizing radiation. Numerous studies have described a very broad spectrum of substances (radioprotectors, radiomitigators, radiomodulators) that are difficult to compare due to the different endpoints used in various studies, radiation doses administered, concentrations used, and the different evaluation procedures used to quantify their protective capacities [8,9,10,11].

In general, these radioprotective substances protect against radiation-induced genetic damage, teratogenic effects, changes in physiological systems, cell death, or against the generation or limiting the effects of free radicals induced by ionizing radiation [1,8]. However, some results of these radioprotective substances have often been apparently contradictory. While some radioprotective substances follow the general behavior pattern of sulfhydryl compounds, losing their protective capacities when administered after exposure to radiation [12,13], other substances significantly increase their protective capacities when administered after exposure to radiation [14,15]. On other occasions, when radioprotective substances are characterized by increasing cell survival of irradiated populations, some of these substances show paradoxical effects, portraying themselves as radiosensitizing agents in different cell lines [15]. Furthermore, substances with antioxidant activities and significant radioprotective capacities against direct exposure to ionizing radiation lose this capacity in bystander cells, i.e., cells not directly exposed to ionizing radiation [16]. Due to inconsistent and sometimes conflicting results, combining multiple agents has been proposed to achieve broader radioprotection than single agents alone [17]. This is possibly due to a lack of precise knowledge of the cellular protection mechanisms involved.

One of the radioprotective substances that shows these discrepancies and even presents paradoxical radiosensitizing effects in some cell lines is rosmarinic acid (RA). RA is a common ester derived from caffeic acid and (R)-(+)-3-(3,4-dihydroxyphenyl) lactic acid with proven medicinal applications [18,19,20]. RA has demonstrated significant radioprotective and genoprotective capacities “in vivo” and “in vitro” against damage induced by ionizing radiation [21,22,23,24,25] and even against damage induced by ultraviolet radiation [23]. This protective capacity has been attributed to its antioxidant capacity, mediated by its ability to eliminate radioinduced free radicals produced in cells directly exposed to ionizing radiation (“target” theory) [23,25].

The study aimed to compare the ability of various antioxidant substances to protect human lymphocytes from ionizing radiation-induced chromosomal damage using the cytokinesis-block micronucleus (CBMN) assay. Based on their chemical properties, we hypothesized that differences in genoprotective efficacy might be partly explained by distinct antioxidant profiles and that antilipoperoxidant activity could represent a particularly relevant, albeit still hypothetical, determinant of protection against radiation-induced chromosomal damage.

## 2. Materials and Methods

### 2.1. Chemicals and Reagents

Vitamins C and E, gallic acid, ellagic acid, eriodyctiol, β-carotene and RPMI 1640, F10, PHA, cytochalasin B, streptomycin, penicillin, phosphate buffered saline (PBS), methanol, heparin, sodium chloride, and sodium bicarbonate were obtained from Sigma-Aldrich (Madrid, Spain). Rosmarinic acid, diosmin, rutin, and quercetin were obtained from Extrasynthese (Genay, France). Apigenin, apigenin-K, carnosic acid, carnosol, 3-hydroxytyrosol, 6-hydroxytyrosol, procyanidins (short (P short), medium (P medium), and long (P long)), olive leaf extract, citrus extract, green tea extract, pomegranate extract, grape seed extract, and chestnut were purchased from Nutrafur S.A. (Alcantarilla, Spain). Amifostine (WR-2721, Ethyol^®^) was from Schering-Plough S.A, (Madrid, Spain). Zoledronic acid (Zometa^®^) was a product of Novartis Pharmaceutica (Barcelona, Spain); dimethyl sulfoxide (DMSO) was purchased from Merck (Darmstadt, Germany) and fetal bovine serum was obtained from Gibco (Life Technologies S.A., Madrid, Spain). Pycnanthus angolensis seed extract (PASE) was prepared in house [24]. Fat-soluble substances were dissolved in DMSO, and DMSO solvent controls were included both before and after irradiation at the same final concentration used for the test compounds to monitor the potential contribution of the solvent. Further details about the composition and characteristics of the extracts used can be found in the additional manuscript file.

### 2.2. In Vitro Micronucleus Assay (CBMN)

**Figure 1:**
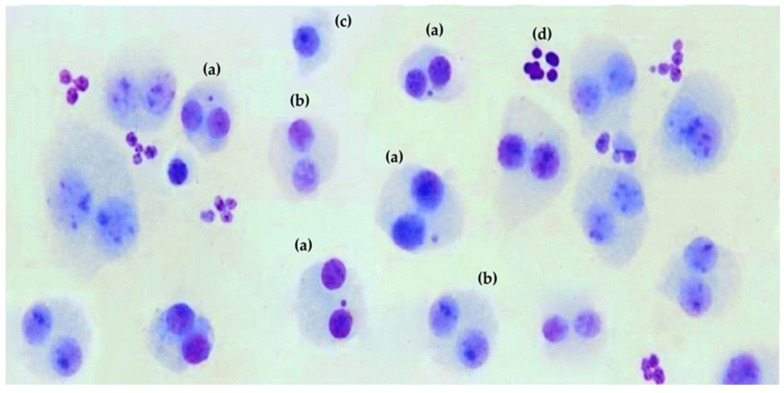
Binucleated cells in irradiated human lymphocytes as seen in the cytokinesis-block micronucleus method (600×): (a) binucleated cell with micronucleus, (b) binucleated cells blocked by cytokinesis, (c) lymphocyte, and (d) erythrocyte remains.

Informed consent was obtained from 6 apparently healthy volunteers whereupon 30 mL of venous blood was drawn from a vein located on the inside of their elbows and kept in heparinized tubes until needed. Samples from all six donors were processed under identical culture, irradiation, and scoring conditions. Donor was treated as a random factor, and data were pooled across donors after confirming the absence of a significant donor-by-treatment interaction. Each independent blood sample from a given donor, processed on a different day and subjected to a defined treatment and exposure setting, was considered one experimental unit. For each condition, five independent experiments were performed on separate culture days using samples from six donors. Test substances were administered prior to ionizing radiation exposure, followed by the cytokinesis-block micronucleus (CBMN) assay, as previously described [26]. The procedure adhered to adapted International Atomic Energy Agency guidelines for biological dosimetry and for screening genoprotective agents against radiation-induced damage [27,28]. The cytokinesis-block proliferation index (CBPI) in control cultures from all donors was 1.65 ± 0.10, confirming an adequate proliferative capacity for the evaluation of micronuclei (MN/500 BC) using the CBMN assay. In all experimental conditions (treatment, radiation alone, and combined treatment), CBPI values remained between 1.3 and 1.7, within the range recommended by OECD Test Guideline [29], indicating that culture proliferative quality was adequate for the correct interpretation of micronucleus results. All the substances tested were dissolved in PBS or DMSO at a concentration of 25 µM, of 25 µL were added to 2 mL of heparinized blood immediately before or after (less than 1 min) gamma radiation exposure. After hypotonic treatment and fixation, cell suspensions were dropped onto clean glass slides and allowed to air dry at room temperature. After 24 h of incubation, slides were stained by sequential immersion as follows: first, in pure May Grünwald stain (Merck, Darmstadt, Germany) for 3 min; then, in a 50% May Grünwald solution (50% May Grünwald and 50% distilled water; Braun, Melsungen, Germany) for 2 min; subsequently, without intermediate washing, slides were immersed in Giemsa stain (Merck, Darmstadt, Germany) for an additional 2 min; finally, they were washed by individual immersion in 100 mM phosphate buffer, pH 6.2, and left to air dry before microscopic evaluation. For each experimental condition, at least 3000 binucleated cells (CBs) were scored by three independent observers in a double-blind using a light microscope, and their results are expressed in MN/500 BC (Figure 1). All test substances were assessed at a single concentration of 25 µM, chosen from preliminary studies as non-cytotoxic. This concentration was also suitable for solubility and practical handling. This design was chosen to allow a standardized comparative screening of genoprotective effects rather than a detailed concentration–response characterization for each substance. For lipophilic compounds dissolved in DMSO, micronucleus frequencies were compared with the corresponding DMSO-only controls. In some analyses, results are shown as DMSO-normalized values, obtained by subtracting the effect of DMSO alone from the total effect of the compound plus DMSO.

### 2.3. Irradiation

The IBL 437C (Schering Cis, Biointernacional, Berlin, Germany) blood irradiator indicated for the irradiation of blood and biological products/samples was used for irradiation. It was equipped with a Cs-137 radioactive source and had an activity of 1700 Ci and a dose rate of 50 mGy/s. Control and treated blood samples were irradiated at ambient temperature and pressure for 40 s to a total dose of 2 Gy. To obtain the dose–response curve, the frequency of MN in 500/BC in samples exposed to 20 different doses of gamma radiation in the range of 0–16.362 mGy was determined. The final radiation dose was confirmed by thermoluminescent dosimeters (TLDs) (GR-200^®^; Conqueror Electronics Technology Co., Ltd., Shenzhen, China).

### 2.4. Statistical Analysis

The increase in the frequency of the appearance of micronuclei in cytokinesis-blocked binucleated cells was analyzed as an expression of the chromosome damage induced by gamma radiation. On the contrary, the reduction in the frequency of micronuclei in treated cells showed the genoprotective capacity of each substance. Data are expressed as mean ± SE of five independent experiments. Group differences were assessed by one way analysis of variance (ANOVA) and, when appropriate, by Tukey’s honestly significant difference (HSD) post hoc test for pairwise mean comparisons, in order to control for multiple comparisons across substances and exposure settings; linear and polynomial regression and correlation analyses were also performed. A two-tailed *p* < 0.05 was considered statistically significant.

Furthermore, two other parameters, namely, magnitude of protection (MP) and dose reduction factor (DRF), were also determined. The MP expresses the protective capacity of a radioprotective substance, again often denoted as a percentage [30], calculated with the formula MP (%) = [(FMN control irradiated − FMN treated irradiated)/(FMN control irradiated)] × 100, where FMN control irradiated is the frequency of micronuclei in the irradiated control samples and FMN treated is the frequency of micronuclei in the samples treated with test substances and irradiated. MP allows the determination of the genoprotective capacity of a substance and comparison of it with the same dose of radiation administered to a control sample treated with the same substance but not irradiated.

The dose reduction factor (DRF) was defined as the ratio between the radiation dose required to produce a given micronucleus frequency in untreated irradiated cultures and the dose that produced the same effect in cultures treated with each test substance [31]. Practically, for each compound, we used the micronucleus frequency observed at 2 Gy in the presence of the substance, interpolated the corresponding dose on the calibration curve of irradiated controls (DFMN2Gy), and calculated the DRF as DRF = DFMN2Gy/DFMN2Gy_treated_. Thus, a DRF > 1 indicates that the test substance reduces the biological effect of a fixed 2 Gy exposure to a level equivalent to a lower dose in untreated cells, reflecting its genoprotective capacity.

## 3. Results

**Figure 2:**
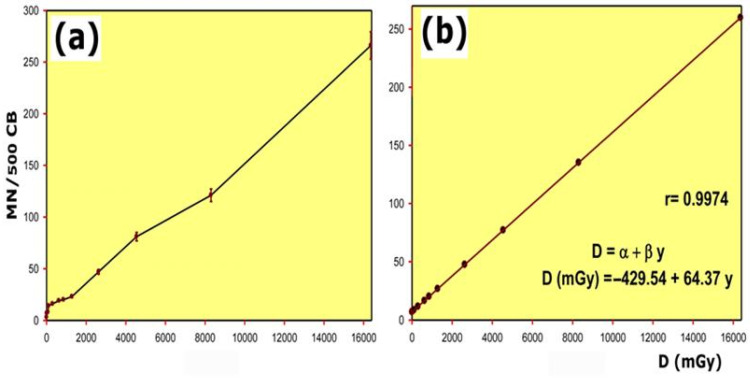
Dose–response relationship of radiation-induced micronuclei in cytokinesis-blocked human peripheral lymphocyte cultures: (**a**) results obtained; (**b**) expected statistical results.

In control cultures from all donors, the baseline frequency of micronuclei remained within the expected range for human lymphocytes in the CBMN assay (MN/500 BC). This is consistent with previously reported values in healthy populations and supports the suitability of our experimental system for assessing chromosomal damage [29]. In our study, the dose–response curve obtained after irradiating blood samples from healthy donors with gamma radiation showed a linear relationship and a reliability of 99.74% within the dose range between 1.3 cGy and 1636 cGy. The conditions used for this study permit the estimation of the radiation dose as a function of the number of MNs produced in cytochalasin-B-induced cytokinesis-blocked human lymphocytes (Figure 2). To estimate the DRF, the dose of gamma radiation necessary to obtain a certain number of micronuclei was obtained from the equation D = −429.54 + 64.37y, where y is the MN frequency determined at 2 Gy when the test radioprotective substance was present and D is the gamma radiation dose (in mGy) required to obtain the same number of micronuclei in untreated cultures.

The addition of 25 µL of 25 µM test substances to the blood did not show statistically significant differences in the amount of MN produced with respect to untreated control cultures. This observation expresses an absence of genotoxic effect when the said test substances were administered to the cell cultures. However, when zoledronic acid (Zometa^®^) was administered directly without dilution, it produced a significant increase in MN frequency compared to control cultures, attaining a count of 35 ± 2 MN/500 BC, showing significant differences (*p* < 0.001) compared to controls, and expressing the genotoxic capacity of zoledronic acid.

**Figure 3:**
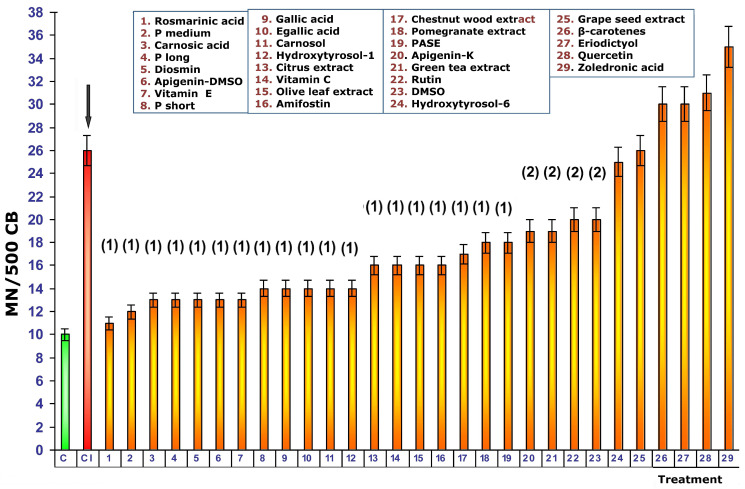
Frequency of micronuclei in cytokinesis-blocked cultures of irradiated human lymphocytes treated with test substances immediately before exposure to ionizing radiation. Data are mean ± SE of four independent experiments (C, Control; CI, irradiated control; black arrow, Control vs. irradiated control (*p* < 0.001); (1), treatment vs irradiated control (*p* < 0.001); (2), treatment vs irradiated control (*p* < 0.01)).

Exposure of untreated control cultures to 2 Gy of gamma radiation produced a significant increase in the frequency of MN attaining a count of 26 ± 2 MN/500 BC. This portrayed significant differences in the frequency of MN occurrence with respect to non-irradiated control cultures (*p* < 0.001) and expresses chromosomal and genotoxic damage induced by ionizing radiation (Figure 3). No significant differences were observed in the frequency of appearance of MN produced by exposure to 2 Gy of gamma radiation and the addition of 25 µL of zoledronic acid used at 100% of its original strength (Zometa^®^), expressing a similar genotoxic capacity between both agents analyzed.

### 3.1. Addition of Test Substances Before Exposure to Ionizing Radiation

Exposure of blood samples treated with the different test substances to 2 Gy of gamma radiation before radiation exposure produced different effects on the frequency of MN appearance in irradiated and cytokinesis-blocked human lymphocyte cultures expressing different degrees of genoprotection (Figure 3). In the first 18 test substances represented in Figure 3, a significant reduction in the expected MN frequency was observed when the treated cells were exposed to ionizing radiation (*p* > 0.001), expressing a significant protective capacity against the chromosome damage induced by ionizing radiation (see numbers 1–19, Figure 3). Among these 19 test substances, the cultures treated with rosmarinic acid stand out, since they showed the greatest reduction in expected MN frequency when the substances were administered before exposure to radiation. The MN frequency in these cultures was similar to that of non-irradiated controls (Figure 3). The next four substances administered before radiation exposure (see numbers 20–23, Figure 3) also show a significant reduction in the frequency of MN compared to irradiated control cultures (*p* < 0.01), which also expresses a significant degree of genoprotection but less intense than the previous substances. The rest of the test substances administered before exposure to radiation do not show any decrease in the frequency of appearance of MN, which expresses a lack of genoprotection. On the contrary, it can be highlighted that quercetin and zoledronic acid both show an increase in the frequency of MN after exposure to 2 Gy of radiation (*p* < 0.01). This observation expresses an increase in the chromosomal damage induced by ionizing radiation in these induced cultures, which could be interpreted as a radiosensitizing effect of these substances (Figure 3).

To address the issue of low solubility in aqueous medium, some fat-soluble substances used were dissolved in DMSO for this reason, and DMSO was tested at the concentrations used to prepare the said solutions.

**Figure 4:**
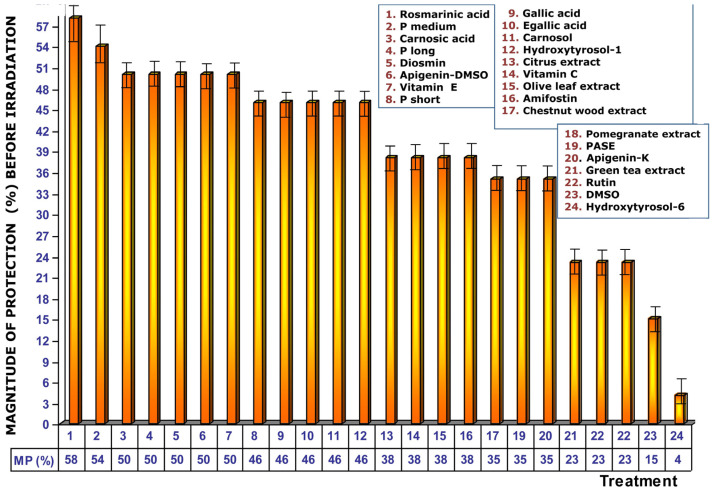
Magnitudes of protection obtained when tested substances are administered immediately prior to exposure to ionizing radiation.

Figure 4 shows the magnitude of protection conferred by the test substances when administered before exposure to ionizing radiation. Under these conditions, RA presents the greatest genoprotective ability with a magnitude of protection of 58%. In this comparative study of radioprotective capabilities of different substances, it is worthy to note that amifostine, the only radioprotective substance clinically approved by the Food and Drug Administration (FDA), expressed a magnitude of protection of 38%; other known radioprotective substances, such as vitamins E and C, elicited magnitudes of protection of 50% and 38%, respectively.

**Figure 5:**
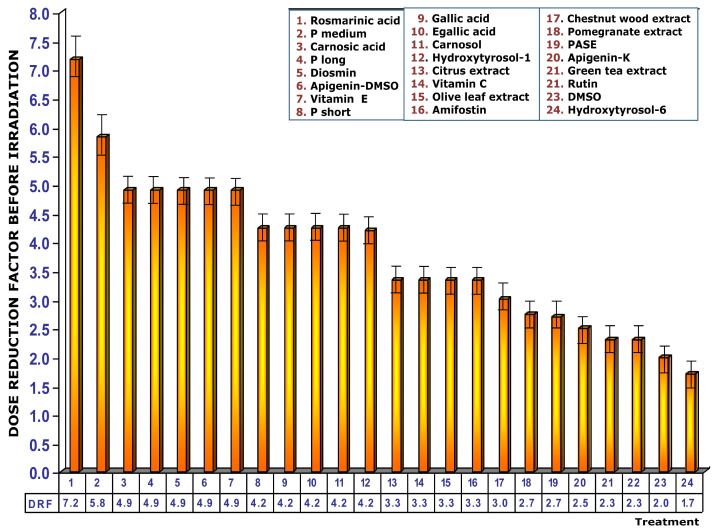
Dose reduction factor (DRF) values obtained upon the addition of test substances to cell cultures immediately prior to ionizing radiation exposure.

Figure 5 shows the dose reduction factors (DRFs) obtained when cultures were administered with different test substances before exposure to 2 Gy of gamma radiation. Using the dose–response module we developed in our study, we calculated the radiation doses (in mGy) required to provoke the formation of MN when the test substances were added to the cultures before radiation exposure. The DRF value of 7.2 obtained with RA deserves some highlight. It is much higher than that obtained for amifostine (3.3), the only radioprotectant currently in clinical use; for vitamin C (3.3); and for vitamin E, a powerful radioprotectant, which has a DRF of 4.9. This DRF reduction determined for RA (7.2) expresses that, after exposure to 2 Gy of gamma radiation, the treated samples express chromosomal damage equivalent to an exposure of 0.27 Gy of gamma radiation.

When the test substances were administered immediately before exposure to ionizing radiation, our results show RA to be the substance with the highest genoprotective capacity of all the substances tested. RA portrayed a frequency of 11 ± 2 MN/500 BC after exposure to 2 Gy of gamma radiation, an MP of 58%, and a DRF of 7.2.

### 3.2. Addition of Test Substances to Cell Cultures After Exposure to Ionizing Radiation

**Figure 6:**
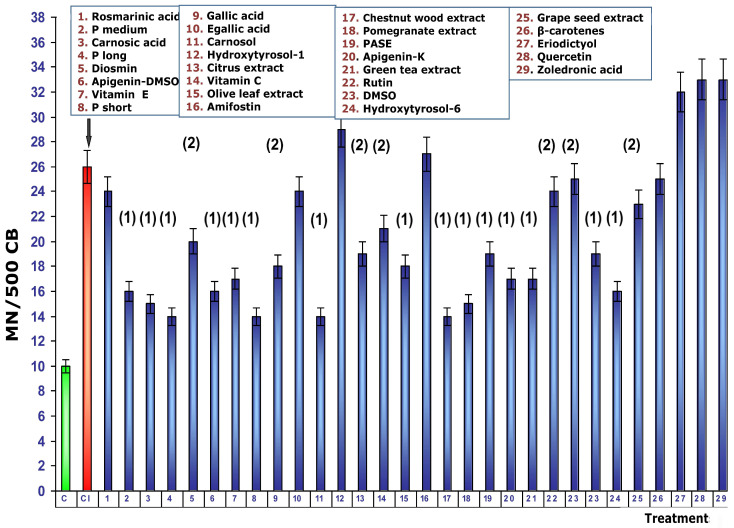
Micronuclei yield in cytochalasin B-blocked irradiated human lymphocyte cultures exposed to test substances immediately after exposure to ionizing radiation. Data are mean ± SE of four independent experiments (C, Control; CI, irradiated control; black arrow, Control vs. irradiated control (*p* < 0.001); (1), treatment vs irradiated control (*p* < 0.001); (2), treatment vs irradiated control (*p* < 0.01)).

Administration of the test substances immediately after exposure to 2 Gy also produced a decrease in the frequency of MN in the majority of the tested substances. However, this reduction is significantly lower than the MN frequencies determined when the substances were administered before exposure to ionizing radiation, which expresses a lower degree of protection against radioinduced chromosomal damage. DMSO and amifostine, as sulfur-containing substances, do not confer any degree of genoprotection, presenting a frequency of MN similar to irradiated control cultures (Figure 6), which expresses a loss of protective capacity that they possessed when present in the samples before exposure to ionizing radiation. In this sense, the loss of the genoprotective capacity of RA is noteworthy, as it does not portray substantial differences in expression of MN frequency with respect to the irradiated control samples. Expressing a significant loss of its genoprotective capacity when it is administered to the samples after exposure to ionizing radiation.

**Figure 7:**
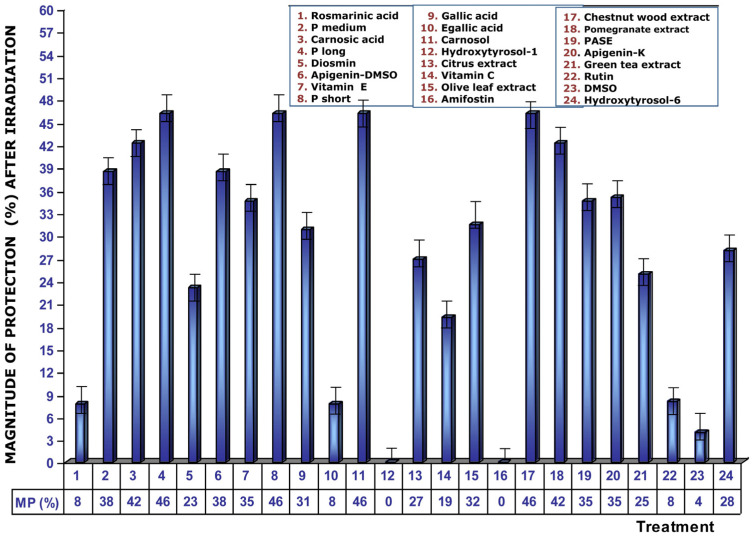
Magnitudes of protection determined when test substances are administered immediately after exposure to ionizing radiation.

The magnitudes of protection determined under these conditions were also observed to be lower, focusing attention on the loss of genoprotective capacities of amifostine (0%), DMSO (4%), rutin, egallic acid (8%), and RA, which practically lost its genoprotective capacity (Figure 7). Procyanidins (P long and P short) and carnosol show the highest MP in these conditions.

**Figure 8:**
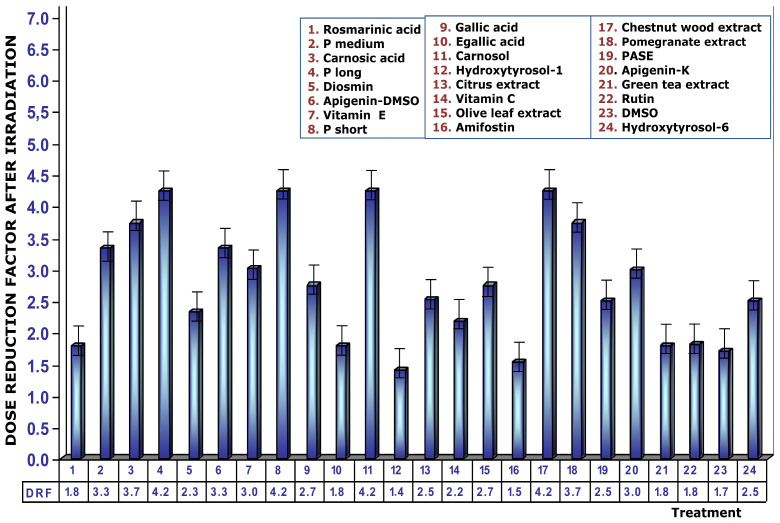
Dose reduction factors (DRFs) obtained upon addition of test substances immediately after ionizing radiation exposure.

Figure 8 displays the DRFs of the substances added to the cell cultures immediately after exposure to ionizing radiation. The values seem to suggest that DRFs obtained are lower than what was determined when these substances are present in the biological environment before the cultures are exposed to ionizing radiation. In this paper, RA shows an MN frequency in irradiated cultures of 24 ± 2 MN/500 BC and a calculated magnitude of protection of only 8% and a DRF of 1.8, which implies a significant decrease (*p* < 0.001) in its genoprotective capacity compared to cultures treated with RA before exposure to ionizing radiation. Under these conditions, long-chain procyanidins (P long) show a DRF of 4.3, which would mean that 2 Gy would produce chromosomal damage equivalent to an exposure of 0.48 Gy.

**Figure 9:**
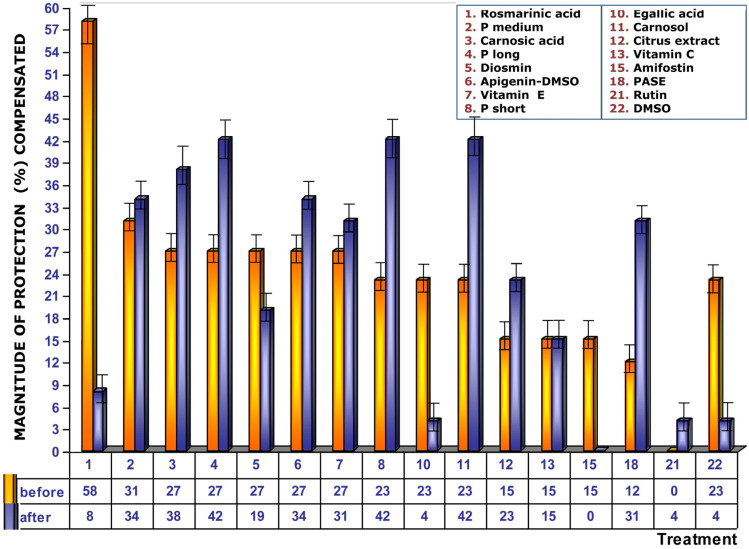
Normalized magnitudes of protection for the test substances after compensating for possible additive effect of the DMSO used to dissolve the test substances in the biological environment. Compensation was performed on water-insoluble substances that were dissolved in DMSO. Normalized results are shown for substances added prior to and immediately after exposure to ionizing radiation.

Figure 9 shows the results of fat-soluble (water-insoluble) substances and extracts tested. These fat-soluble substances were dissolved in DMSO to improve their cellular availability at non-cytotoxic concentrations of DMSO. In this study, DMSO was included for two reasons: firstly, as a sulfur-containing antioxidant compound with a possible genoprotective capacity; and secondly, to analyze for a possible additive effect with the substances in which the test substances were dissolved. The study revealed that DMSO only shows a significant genoprotective effect when found in the biological environment before exposure to ionizing radiation but has no effect when administered after exposure to ionizing radiation. Therefore, when DMSO is used to dissolve fat-soluble substances, it could be assumed that, with these substances, an additive or synergistic effect may occur between the genoprotective capacities of DMSO and the fat-soluble substance tested. Figure 9 shows the magnitudes of protective effects (MPs) of fat-soluble substances dissolved in DMSO after compensating for the genoprotective effects induced by DMSO in the cultures when they were added both before and immediately after ionizing radiation exposure. The results of the magnitude of protection observed for RA are not affected under these circumstances. However, under these conditions, it is observed that fat-soluble substances present, in general terms, greater genoprotective capacities when administered immediately after exposure to ionizing radiation, modifying the previous perception shown in Figure 4.

The results of other fat-soluble substances dissolved in DMSO (quercetin, eriodyctiol, and beta-carotene) are not shown since they lack genoprotective capacities and even behave as radiosensitizing substances. Their results may also be confounded by the genoprotective effects of DMSO used to dissolve them.

## 4. Discussion

CBMN has been used as an analytical tool to evaluate chromosomal damage and genomic instability caused by DNA-damaging physical and chemical agents. It has also been used extensively to evaluate cytogenetic damage among professionally exposed workers and during occupational accident scenarios, including DNA damage induced by ionizing radiation. In the cytokinesis-block micronucleus assay, MN may originate from either acentric chromosome fragments or whole chromosomes that fail to properly segregate, thus reflecting both clastogenic and aneugenic events. In the specific context of ionizing gamma radiation, the MN detected are expected to arise predominantly from DNA double-strand breaks and acentric fragments, although a minor aneugenic component cannot be excluded [29]. The practical utility of the MN assay for genotoxicity assessment has been approved by credible international organizations, including IAEA, OECD, and OIN [32]. The CBMN assay is a relatively simple and widely used technique for the evaluation of genotoxically induced genetic instability [27,28]. The micronucleus is a part or a fragment of a chromosome that remains outside the spindle due to a chromosome segregation error during mitosis and remains isolated in the cytoplasm after cell division. This is why it is observed as one or more intracytoplasmic inclusion body(ies) clearly separated from the nucleus, with chromatic characteristics similar to those of the cell nucleus but of a significantly smaller size. These structures are considered remnants of genetic material that have not been able to be incorporated correctly into the daughter cells (Figure 1). Micronuclei reflect chromosomal aberrations produced by chromosomal breaks produced by errors during DNA replication (mostly due to double strand breaks (DSBs) that are revealed after cell division). A basal or spontaneous MN frequency can be determined in normal populations and increases significantly after exposure to genotoxic agents or mutagenic substances [28]. The CBMN assay was used to assess X-ray-induced genotoxicity and to screen substances for genoprotective effects, with a reduced number of micronuclei indicating decreased radiation-induced chromosome damage [28].

In our study, we established a linear relationship between the doses of gamma radiation used and the frequency of appearance of micronuclei. Numerous studies have also described a similar relationship [33,34,35,36,37,38,39]. Some studies have also established a linear-quadratic relationship between the dose of gamma radiation used and the frequency of MN [40,41,42,43,44]. These differences have been attributed to different causes: the type of ionizing radiation used, the different biological effectiveness of the radiations used [44,45], the cell culture time [39], and different technical modifications used and even differences in the criteria for the identification of the MNs used in the different laboratories [46].

The spontaneous or basal frequency of micronuclei is taken into account in developing the dose–response relationship. The spontaneous frequency of micronuclei has been decreasing as the assay has evolved methodologically. A baseline frequency of 10–15 MN/500 BC was considered normal when the micronucleus test was used without cytokinetic blockade, while, when the test was performed with blockade using Cytochalasin B, the baseline or spontaneous frequency decreased to 5–10 MN/500 BC [28,37,44,45]. In our study, the spontaneous or baseline frequency determined in non-irradiated peripheral blood lymphocyte cultures was 0.02 MN/BC. This value of spontaneous frequency is similar to that observed by some authors [44,47] and somewhat higher than that described by others [26,36,38,46]. To minimize the influence of this parameter, most of the studies reviewed recommend performing the micronucleus count on 500 blocked cells [26,27,28,48]. In our study, the spontaneous frequency found is similar to that described in different previous studies [26,44].

We studied amifostine and DMSO as classic radioprotective reference substances since they are sulfur-containing substances with sulfhydryl bonds (SH-SH) and were among the first structures described in radiobiology to attenuate radiation-induced damage [12,31,49]. Today, amifostine is the only substance used in clinical settings, although its application is limited to the protection of the salivary glands to reduce the side effects of radiotherapy in the treatment of head and neck cancers [50,51,52,53]. The precise mechanisms by which they exert their radioprotective actions are not completely clarified. Three levels (mechanisms) of action have been suggested: at the molecular level, its ability to scavenge free radicals by its proficiency to donate hydrogen and bind to biological targets to form disulfide compounds; at the physiological level, its ability to induce hypoxia, hypothermia, or shock; and, at the organic level, its ability to stimulate the recovery of irradiated cell populations [12,13]. Based on their chemical structure, it has been described that these sulfur-containing substances have greater radioprotective capacities due to the presence of the following five chemical features: the presence of a sulfhydryl or thiol group (-SH) protected by a phosphate; 2 or 3 C atoms separating the amino group from the sulfur atom; the presence of a second amino group separated from the first by 3-C atoms; high water solubility (i.e., high pKa value); and, finally, a protective capacity at lower concentrations [54]. In this study, amifostine showed medium genoprotection, behaving like a classic radioprotector since it only protects when it is in the biological environment before exposure to ionizing radiation. Previous references regarding the genoprotective capacity of amifostine administered after exposure to ionizing radiation using the CBMN assay are not available. However, when administered before irradiation, Kopjar et al. (2006) [55] described a radioprotective effect of amifostine using the CBMN assay; and Müller et al. (2004) [56] also determined its genoprotective capacity under these conditions, although using the Comet assay. Moreover, DMSO is an antioxidant substance with a high capacity to eliminate hydroxyl radicals in vitro [57,58]. In this study, DMSO showed a lower genoprotective capacity when compared with amifostine, which could be explained by the absence of some chemical features enumerated above [55]. Unfortunately, the most radioprotective doses of these substances are usually toxic to the cell and only show genoprotective capacity when present in the biological environment before radiation exposure [21,22,54]; so, they have no ability to reduce the cascade of free radicals produced after exposure to ionizing radiation.

Our results confirm the “in vitro” genoprotective capacity of vitamins E and C regardless of whether they are administered before or after irradiation, in a similar way to previously published studies. In this study, vitamin E shows a higher degree of genoprotective capacity than vitamin C, similar to that described in previous in vivo studies [22,29]. Initially, this greater protective capacity of vitamin E was attributed to the increase in the activity of the immune system in vivo, given that both vitamins have antioxidant capacities and are free radical scavengers [30]. Subsequently, it has been suggested that the peroxyl radicals generated “in vitro” are neutralized more efficiently by tocopherol than by ascorbic acid [9].

In this study, the protective effect of a possible lipid component is observed in the genoprotective capacity of hydroxytyrosol-1, a water-soluble substance in comparison with hydroxytyrosol-6. Hydroxytyrosol-1, a phenolic compound with a lower molecular weight and higher water solubility, shows genoprotective tendencies when present before radiation treatment but loses protective activity when added post-radiation. In contrast, hydroxytyrosol-6 maintains its genoprotective capacity even when administered after exposure to radiation, showing significant differences compared to hydroxytyrosol-1. We have previously established that compounds with different chemical structures possess different genoprotective capacities. Even though there is no single chemical structure that is a protagonist of this genoprotection, a direct relationship can be seen with the antioxidant capacities of the substances tested. In this study, we tested flavonoids with similar chemical structures that showed very varied radioprotective responses, while some showed intense genoprotection (procyanidins); on the contrary, others showed a significant increase in their genotoxicity (quercetin). Within flavonoids or polyphenols, the flavan-3-ol structure shows the highest genoprotective capacity [59,60,61,62]. The high antigenotoxic activity of procyanidins can be explained by their chemical structure, due to the presence of high levels of conjugated double bonds between the catechol groups in the B ring and the free 3-OH groups of the polymeric polyphenolic skeleton, in addition to the stability of the flavonoid aroxyl radical generated during the processes [4,16,59,60].

Three substances extracted from rosemary (carnosic acid, carnosol, and RA) constituted the major radioprotectors in our study. The presence of a catechol group in the aromatic ring (C11–C12) of the phenolic diterpene backbone of rosemary is probably the most important structural element accounting for the antioxidant activity of these compounds. According to the data shown by carnosic acid and carnosol, the presence of a free carboxylic group in this diterpene skeleton increases radioprotective activity [13,15,16,25].

In our work, to be able to administer fat-soluble substances to cells, we used dimethylsulfoxide (DMSO), an organosulfur compound with antioxidant capacity for dissolution. Thus, a possible additive effect between the genoprotective effect of DMSO and the genoprotective effect of the substances that were solubilized in DMSO should be evaluated. The importance of this factor is reflected in our results obtained regarding the genoprotective capacity of apigenin. Different authors have described the radioprotective capacity of apigenin, a water-insoluble flavonoid, which has been considered a potent radioprotectant and whose results are in agreement with the results from apigenin dissolved in DMSO used in this study (apigenin-DMSO) [62,63,64,65]. However, we also used the potassium salt of apigenin (apigenin-K) that is more soluble in water and permits direct solubilization in water without having to use DMSO for its dissolution. We determined differences in the genoprotective effects of both substances. A significant decrease in the genoprotective effect of water-soluble apigenin-K was observed, which reflects an additive genoprotective effect of DMSO when used to dissolve apigenin.

Due to the fact that DMSO lacks genoprotective capacity when administered after exposure to ionizing radiation, it is possible to determine the genoprotective capacity of the tested substances dissolved in DMSO and that significantly modified all the results obtained in the treatments administered before exposure to ionizing radiation. Surprisingly, if the results are recomposed by eliminating this additive genoprotective effect contributed by the solvent (DMSO), a significant change is observed in the genoprotective capacity of the substances tested. A limitation of our study is the potential confounding role of DMSO in cultures treated with lipophilic antioxidants. All fat-soluble compounds were dissolved in DMSO, and we observed that DMSO alone exhibited a modest genoprotective effect when present before irradiation. For this reason, lipophilic substances were always compared with the corresponding DMSO-only controls, and in some cases, their effects were expressed as DMSO-normalized values. Nevertheless, particularly in post-irradiation treatments, part of the apparent protection observed with certain lipophilic compounds may still reflect, at least in part, the behavior of the solvent itself rather than the intrinsic activity of the test substance. Thus, the differences between water-soluble and fat-soluble antioxidants in this setting should be interpreted with caution, and future studies employing alternative solvents or solvent-free formulations will be required to disentangle the specific contribution of DMSO from that of the compounds evaluated.

In contrast to the initial assessment, a higher genoprotective capacity against radiation-induced genotoxic damage is observed in the substances administered after exposure to ionizing radiation (Figure 9). These results indicate that the significant protection of the genetic material can be achieved even if they are administered after exposure to ionizing radiation. This reflects that the greatest genotoxic damage produced by ionizing radiation is produced by radioinduced lipid peroxidation. It could also indicate that reduction in genotoxic damage could be achieved if these substances are administered even up to one hour after exposure to ionizing radiation.

These results contradict established phenomena in classical radiobiology since the harmful effect induced by ionizing radiation could be minimized even by administering some radioprotective substances after exposure. As proposed by Pincemail et al. (1986) [64], the fact that the genoprotective capacities of these types of substances vary depending on the time of administration with respect to radiation exposure could be explained by differentiating between antiradical activities (against superoxide anion and hydroxyl radicals) and antilipoperoxidant activities (against lipoperoxy radicals).

Due to the discrepancy in the results obtained by a substance that can lose its genoprotective capacity when administered after exposure to ionizing radiation (RA, DMSO, Amifostine), present similar capacities when administered before or after exposure (CA, COL, Vit C) or even be masked by the additive effect of the DMSO solvent used (for fat-soluble substances tested), different studies propose mixtures of genoprotective substances or extracts of antioxidant substances, with the intention of obtaining a broad-spectrum genoprotective mixture [12,17,66,67]. Obviously, it is necessary to know the different individual mechanisms of action of each substance so that we can obtain a cocktail with broad spectrum genoprotection by mixing substances with anti-radical activity (against superoxide anion and hydroxyl radicals) and antilipoperoxidants. In this case, the choice of usage will be informed depending on whether exposure to ionizing radiation has already occurred, as occurs in accidental exposures [16,22,59].

In this study, RA presented the greatest magnitude of protection among all the substances evaluated, and this has been related to its ability to eliminate hydroxyl radicals by having a combination of a ring system that is conjugated with double bonds in its polyphenolic skeleton predominantly the o-dihydroy-phenol or catechol structure. The presence of two catechol groups conjugated with a carboxylic acid functional group improves its antioxidant activity in aqueous medium [16,23,25], presenting the high genoprotective capacity, possibly due to its ability to eliminate antiradical activity (superoxide anion vs. hydroxyl radicals). However, it practically loses this genoprotective capacity when it is administered after exposure to ionizing radiation, when hydroxyl radicals no longer exist in the aqueous medium and portray an inability to eliminate the radicals produced during lipid peroxidation that prolong the genotoxicity induced by ionizing radiation.

RA is an example of radioprotective substances for which contradictory and even paradoxical radioprotective effects have been observed. On the one hand, the loss of radioprotective capacity of RA has been observed both “in vivo” and “in vitro” in the radiation-induced bystander effect (RIBE). In general, ionizing radiation produces a number of different radicals in and around DNA molecules [17,24,34]. Their high reactivity produces an immediate reaction within proximities of their generation [31,49]. These ROS have a very short half-life and can directly damage irradiated cells but could also be eliminated or reduced by RA [31]. However, ROS produced is massive, and the harmful cytotoxic effect will no longer remain only in a localized environment of immediate vicinity of ionization but can also cascade through the reactive species and other free radicals within the intracellular and extracellular environments. This increases interaction with cell membrane phospholipids and initiates lipid peroxidation processes, which tend to increase oxidative DNA damage at a further distance and even hours after direct exposure to ionizing radiation. The half-life of long-lived peroxides is greater than 24 h; so, they have the possibility of exiting the irradiated cells to non-irradiated recipient cells, causing damage to them. For such radicals produced by the lipid peroxidation processes, the administration of RA would lack a neutralizing capacity [14,21]. This surprising loss of radioprotective capacity of RA in the radiation-induced bystander effect seems to be related to its inability to limit lipid peroxidation, which is evidenced by the lack of protection of RA when administered after exposure to ionizing radiation [16].

Elsewhere, a potentially paradoxical effect of RA has been described since, being a potent radioprotectant in most cell lines, it showed a paradoxical radiosensitizing effect in melanoma cells [25]. In normal and tumor prostate cells, RA increases the endogenous production of glutathione, leading to a significant increase in the GSH/GSSG ratio when administered before exposure to radiation. RA and intracellular glutathione together contribute to eliminating radioinduced free radicals after exposure to ionizing radiation. In contrast, none of these protective effects are found in metastatic B16F10 melanoma cells. It has been suggested that, in B16F10 metastatic melanoma cells, the administration of RA possibly activates the metabolic pathway of eumelanin synthesis that would lead to the consumption of intracellular GSH, resulting in the reduction in the GSH/GSSG ratio before radiation exposure. This will cause a reduction in available cellular glutathione, thus reducing protective capacity against radiation-induced oxidative stress. Although RA as an antioxidant maintains its radioprotective activity, stimulation of the metabolic pathway of pheomelanin synthesis causes a decrease in available intracellular glutathione and a paradoxical radiosensitizing effect is depicted with the lower cell survival of these cells [25].

Clinically, the genoprotective capacities of these substances are a function of their concentrations, degree of polymerization, and solubility since these factors affect their bioavailability [17,25,61,62]. Furthermore, the different protection mechanisms are related to their chemical structure and the time of radiation exposure. But it also depends on the properties and metabolic pathways of the tested substance. For patients undergoing diagnostic procedures with controlled exposure times, non-toxic, water-soluble dietary antioxidants with high bioavailability, such as rosmarinic acid (RA), can be administered. These antioxidants may reduce the consequences of radiation-induced genotoxic effects [14]. However, in accidental or unscheduled exposures, RA has no protective effects when exposure to ionizing radiation has already occurred. Other protective agents against lipoperoxidation, such as carnosic acid, carnosol, vitamin E, and procyanidins, should be selected. These same substances would be suitable if the aim is to reduce radiation-induced bystander effect in non-irradiated receiver cells, since RA would not achieve any degree of protection. It is advised that, during radiotherapy, these antioxidant supplements should not be used since they could protect both normal and tumor cells [22]. However, the possibility of using substances that protect healthy cells while exclusively injuring neoplastic cells, as in the case of RA with B16F10 melanoma, would remain open [25]. Finally, given the apparent zero toxicity of many of these substances, which are common components of the human diet, they could also be intentionally incorporated into the diet to reduce the risks of injury in workers professionally exposed to ionizing radiation regardless of the doses of ionizing radiation to which they are at risk of being exposed [21]. At this time, a possible improvement in the genoprotective response induced by the simultaneous administration of a mixture of two or more substances than those tested “individually” in this study remains to be studied. These mixtures could reflect a possible synergy in their protective capabilities and would allow the possible use of a mixture of radioprotectants depending on when the radiation exposure occurs. Different mixtures of radioprotective substances could be used depending on the desired purpose, administered before irradiation exposure to protect against planned exposures, or immediately after accidental exposure to reduce radiation-induced undesirable effects.

A limitation of our study is that all compounds were tested at the same nominal concentration (25 µM), even though differences in solubility, stability, cellular uptake, and intrinsic potency mean that this concentration does not correspond to the same biologically effective exposure for all substances. Consequently, the present data should be interpreted primarily as a comparative screening under standardized conditions, and future studies will be needed to establish full dose–response relationships and potency rankings for selected compounds. The paradoxical, and in some cases even radiosensitizing, effects observed for certain antioxidants in our panel likely reflect a complex interplay between several factors, including the redox context of the system, the timing of administration (before versus after irradiation), and the fixed nominal concentration used (25 µM). Other contributing factors include the use of DMSO as a solvent for lipophilic compounds and the intrinsic pro-oxidant potential of some antioxidants under specific conditions. It is well documented that various polyphenols and other redox-active molecules can switch from antioxidant to pro-oxidant behavior at higher concentrations or in the presence of transition metals, thereby enhancing rather than attenuating oxidative damage and DNA lesions. Such context-dependent redox behavior, together with possible solvent-related effects, may help explain why some substances exhibited clear genoprotective effects, whereas others showed neutral or even radiosensitizing responses in the CBMN assay. Overall, our comparative data supports the view that antilipoperoxidant activity may be an important contributor to the genoprotective effects observed, but this interpretation should be regarded as tentative given the lack of direct mechanistic endpoints in the present study. Future work incorporating specific biomarkers of lipid peroxidation and oxidative stress will be essential to confirm this hypothesis and to further dissect the relative contribution of different antioxidant mechanisms to radiation-induced chromosomal damage. This work is based on an in vitro/ex vivo CBMN assay in lymphocytes from a limited number of donors and does not include complementary mechanistic endpoints; therefore, the results should be interpreted as hypothesis-generating rather than definitive and have limited direct clinical extrapolation.

## 5. Conclusions

We highlight the application of the cytokinesis-block micronucleus (CBMN) assay in irradiated human lymphocytes as a useful tool for screening genoprotective substances against ionizing radiation-induced chromosomal damage. In this experimental setting, rosmarinic acid (RA) showed the highest genoprotective capacity among all substances tested, but only when present in the biological environment before exposure, thus behaving as a classical radioprotective agent. Our comparative data are consistent with the notion that antilipoperoxidant activity may represent a particularly relevant determinant of protection against radiation-induced chromosomal damage, suggesting that lipo-antioxidant substances could be especially effective in safeguarding genetic material from oxidative injury; however, this interpretation remains tentative and should be confirmed in future studies using direct mechanistic endpoints. At a practical level, different combinations of radioprotective substances could potentially be tailored to the intended use, either administered before irradiation to mitigate planned exposures or shortly after accidental exposure in order to reduce radiation-induced undesirable effects.

## Data Availability

The original contributions presented in this study are included in the article and in the Supplementary File. Further inquiries can be directed to the corresponding author.

## Supplementary Materials

The following supporting information can be downloaded at: https://www.mdpi.com/article/10.3390/cimb48040364/s1.
