# Supplementary material for: Different Effects of Antioxidants Against Ionizing Radiation: An Experimental Model of Micronuclei
Source: Curr Issues Mol Biol. 2026 Mar 31;48(4):364. doi: 10.3390/cimb48040364 (PMC13114887; doi:10.3390/cimb48040364)
Supplement: Supplementary file 1 [file cimb-48-00364-s001.zip › cimb-4190027-supplementary.pdf]

Additional information on some extracts used in this study

The following extracts were obtained from Nutrafur S.A. (Alcantarilla, Spain).:

• 1.Soluble Citrus Extract or CE-50.

• 2. Olive Leaf Extracts.

.• 3. P short.

• 4. P long.

• 5. Grape Seed Extract.

• 6. Extracts of *Pomegranate*

.• 7. Chestnut Wood Extract..

• 8 Green Tea Extract.

• 9. Hydroxytyrosol (1 and 6).

1. Soluble Citrus Extract

Soluble citrus extracts (Nutrafur S.A., Alcantarilla, Murcia) is characterized by high concentrations of flavonones and flavones and was obtained from immature fruits of three varities of Citrus fruits growing in the Region of Murcia, an Autonomous Community of Spain, namely: *Citrus limonia* (lemon), Citrus sinensis (sweet orange) and *Citrus aurantium* (bitter orange).

The immature fruits were pulverised and extracted in a water-methanol solvent (20:80, v / v) for three hours at room temperature in a ratio of 5% weight / volume To extract the flavone-like compounds.The extract (suspension) was filtered and the polyphenolic hydro-alcoholic solution obtained concentrated under vacuum in a rotary evaporator, at a maximum temperature of 55 ºC until the methanol was eliminated. Subsequently, the aqueous concentrate was cooled gradually with continous stirring for 24 hours to crystallize the flavonoid compounds from the extract.. The suspension was filtered to obtain a solid which was washed with water and vacuum dried at a maximum temperature of 55 ºC.. Finally, a light brown powdery solid, which we called Citrus Extract was obtained and kept untill required for the assays described in this manuscript. Subsequently, high performance chromatographic analysis (HPLC) was performed on the citrus extract. For this, the extract obtained was dissolved in DMSO at a concentration of 2 mg/ml, and filtered through 0.45 micron nylon membrane to sterilise. A C18 LiChrospher 100 column (250 x 4 mm id) with an average particle size of 5 microns was used as stationary phase. The temperature was 30 ºC, the flow was 1 ml / min and the wavelength used was 280 nm for flavonones and 340 nm for flavones. As phases we use: (A) acetic acid: water (1:99), (B) methanol and (C) acetonitrile. We use a method composed of isocratic stages and a linear gradient.

Flavonones are the most abundant flavonoids in the citrus extract comprising naringin, hesperidin and neohesperidin; however, other flavonones opresent such as eriocitrin and neoeriocitrin and other flavones are noteworthy in as their presence could be very significant influence in reference potential biological activity. Likewise, luteolin a flavone glucoside, two diosmetin flavones (diosmin and neodiosmin) and rhoifolin a neohesperidoside derivative of the flavone apigenin are present.. As with the other water insoluble flavonoids, CE-50 was dissolved in DMSO at a concentration of 0.5 mg/ml. Table S1 and Graph S1 shows the absolute amounts of each of the main flavonoids present in CE-50.

Peak	Polyphenols	T_R (min.)	%
1	Eriocitrin	8.24	1.72
2	Neoeriocitrin	10.12	1.25
3	Luteolin-7-O-rutinoside	11.56	0.60
4	Luteolin-7-O-neohesperidoside	12.42	0.41
5	Isonaringin	15.23	1.10
6	Naringin	16.89	10.76
7	Hesperidin	19.96	6.68
8	Neohesperidin	22.35	7.65
9	Rhoifolin	25.31	1.62
10	Diosmin	30.21	0.87
11	Neodiosmin	36.52	0.56
12	Didimin	52.23	0.25

Table S1. Retention times and absolute percentages of main flavonoids of Soluble Citrus Extract.

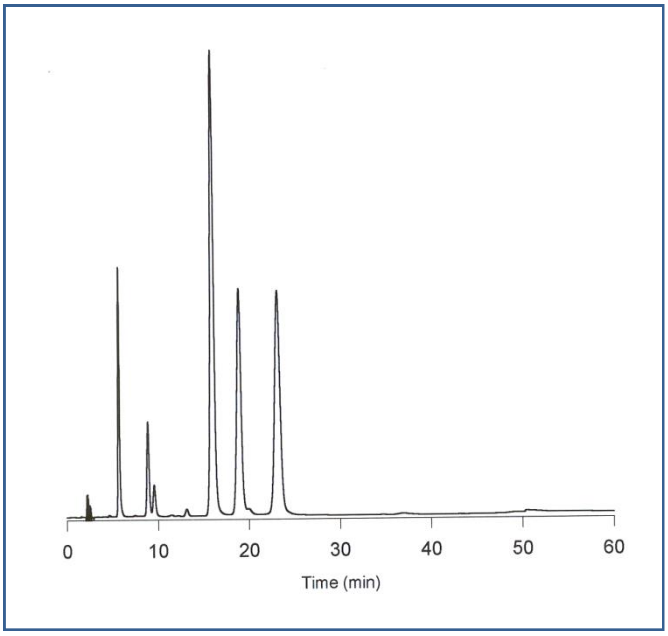

Graph S1. Chromatographic analysis of the components of Soluble Citrus Extracts

2. Olive Leaf Extracts

Olive leaf extract (Nutrafur S.A., Alcantarilla, Murcia) is characterized by the presence of secoiridoid and flavones compounds. It was obtained from leaves of five Olive cultivars (Olea europaea L) species: Villalonga, Alfafarenca, Picual, Cornicabra and Blanqueta.

The dried leaves of the various olive cultivars mentioned were ground and extracted for one hour at room temperature in water-methanol solvent (40:60, v/v) at a ratio of 10% weight/volume. The extract was filtered and the resulting hydroalcoholic filtrate was concentrated in vacuum at a maximum temperature of 60° C in a rotary evaporator to remove the methanol. The resulting aqueous solution was cooled and allowed to settle at room temperature for 24 hours, in order to separate water-insoluble gelatinous materials from water soluble componets by decantation and subsequent filtration. The decanted aqueous solution was filtered through a cellulose and silica gel support to obtain a completely "clearified" final solution. This filtered solution, was concentrated to dryness under vacuum in a rotary evaporator at a maximum temperature of 60 ºC this yielded a crystalline yellowish-brown solid which was called olive leaf extract, and which was saved for conducting corresponding tests described in the manuscript.

Chromatographic analysis (HPLC) of the olive leaf extract was carried out as follows: the extract was dissolved in DMSO, at an analytical concentration of 5 mg/ml and filtered through a 0.45 micron nylon membrane . The HPLC equipment used was a *Hewlett Packard (HP) 1100 Series*

HPLC System, equipped with a diode array detector. A C18 LiChrospher 100 column (250 x 4 mm id) with an average particle size of 5 microns was used as stationary phase. The operating temperature was 30 ºC, the flow rate was 1 ml / min and the wavelength used was 280 nm. The following were used as mobile phases: (A) acetic acid: water (2.5: 97.5) and (B) acetonitrile and the linear gradient method was emplyed for elution. The absolute content of the main polyphenolic compounds present in the olive leaf extract determined by HPLC is shown in Table S2 and Graph S2 of which five types of compounds stand out: secoiridoids, also called oleuropeosides (oleuropein and verbascoside); flavones (luteolin-7-O-glucoside, apigenin-7-O-glucoside, diosmetin-7-O-glucoide, luteolin and diosmetin); flavanols (routine); flavan-3-ols (catechin) and substituted phenols (tyrosol, hydroxytyrosol, vanillin, vanillic acid, and caffeic acid). The most abundant compound in olive leaf extract was found to be oleuropein, followed by hydroxytyrosol, the glucosides of the flavones luteolin and apigenin, and verbascoside. Hydroxytyrosol is a biosynthetic precursor of oleuropein and verbascoside is a glycosylated conjugate of hydroxytyrosol and caffeic acid. Olive leaf extract being soluble in water was administered by dissolving it in water at a rate of 0.5 mg/ml.

Peak	Polifenoles	T_R (min.)	%
1	Hydroxytyrosol	4.8	1.46
2	Tyrosol	5.83	0.71
3	(+)-Catechin	8.41	0.04
4	Caffeic acid	11.56	0.34
5	Vanillic acid	14.17	0.63
6	Vanillin	14.79	0.05
7	Rutin	17.22	0.05
8	Luteolin-7-O-Glycoside	18.10	1.38
9	verbascoside	20.06	1.11
10	Apigenin-7-O-glycoside	21.28	1.37
11	Diosmetin-7-O-glycoside	21.95	0.54
12	Oleuropein	22.76	24.54
13	Luteolin	28.61	0.21

Table S2. Retention time and absolute content by HPLC of the main polyphenolic compounds present in the olive leaf extract

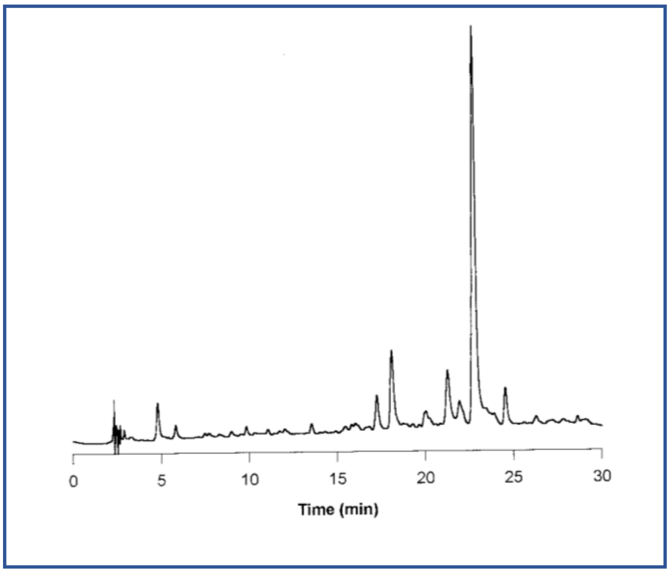

Graph S2. Chromatographic analysis of olive leaf extract.

3. P short

P short (Furfural Español S.A., Alcantarilla, Murcia) is another flavan-3-ol (procyanidin) type compound derived from grape seeds. It was obtained from seeds of four different varieties of *Vitis vinifera*, which were selected from different areas of the Autonomous Community of the Region of Murcia: Macabeo and Airén are white grape varieties and Tempranillo and Monastrel are red grape varieties. To obtain P short, polyphenolic compounds were extracted from ground grape seeds in an atmosphere nitrogen at room temperature for one hour using a water-methanol mixture (25:75, v/v) at a ratio of 10% weight/volume. The extract was filtered and evaporated under vaccum in a rotary evaporator at a maximum temperature of 40 ºC to obtain a reddish-brown solid that was considered as a crude extract of polyphenols and and labelled P short. For addition of P short to blood samples, a dilution of 0.15 mg/ml was prepared in DMSO. In P short extract, polymers with ≥ C4 units (catechin polymers of more than 4 molecules) are the group of flavan-3-ols (procyanidins) present in the highest concentration (66.51%). With respect to the other compounds, the monomers are the most abundant flavan-3-ols; (+) - catechin (8.51%) is more abundant than (-) - epicatechin (6.71%). The rest of procyanidins present in appreciable amounts in this extract corresponded to dimers in which the monomeric units are linked mainly by the C4-C8 interflavan type bonds; the main ones being B4 (2.85%) and the derivatives esterified with gallic acid in position 3 of dimer B1 (3.04%), followed by gallic acid (1.96%), dimer B3 (1.48%), dimer B2 (0.98%) and dimer B1 (0.74%) (Table S3, Graph S3).

Peak	Flavan-3-ols	T_R	% Relative value of P55
G	Gallic acid9.7		1.96
1	B3 (Dimer)	34.3	1.48
2	(+)-Catechin	37.6	8.51
3	B1 (Dimer)	40.6	0.,74
4	T2 (Trimer)	45.4	0.12
5	B4 (Dimer)	49.9	2.85
6	B2 (Dimer)	54.0	0.98
7	B2-3′-gallate	59,1	0,07
8	(-)-epicatechin	62.9	6.71
9	B1-3-O-gallate	64.4	3.04
10	C1 (trimer)	67.4	0.82
11	Polymers ≥ C_4 Units	74.4	66.11
Otros	Other Flava-3-ol dimers and trimers		

Table S3. Relative distribution of main flavan-3-ols present in P short by HPLC.

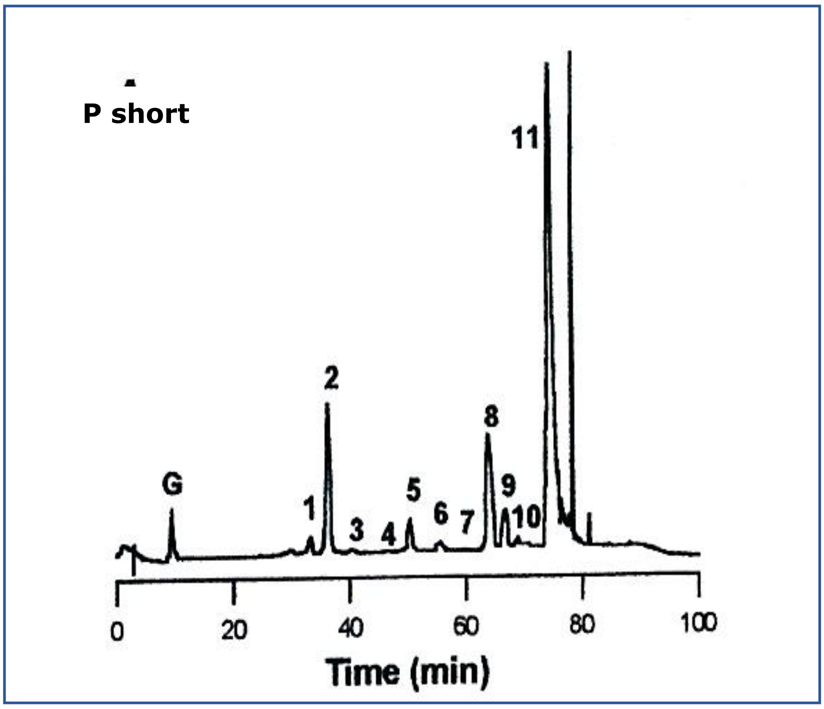

Graph S3. Chromatographic analysis of P short.

4. P long

P long (Furfural Español S.A., Alcantarilla, Murcia) was obtained from P short. The starting material was washed with n-hexane in order to eliminate all the fat-soluble substances of a non-polyphenolic nature characteristic of this plant material. Next, the solid obtained was vacuum-dried until the solvent used is completely eliminated. Subsequently, the solid was suspended for 6 hours in water at a concentration of 5% weight /volume to separate the high molecular weight polyphenolic compounds from the water soluble fractions. The insoluble material was then filtered and dried under vacuum at a maximum temperature of 40 ºC, obtaining a dry powdery red solid, which we call P long. Before adding it to blood samples, P long was dissolved in DMSO at a concentraion of 0.15 mg of per ml of DMSO. In the P long extract, the polymers with ≥ C4 units constitutes practically the only group of procyanidins present in the extract (99.45%) with only a small amounts of (+) - catechin (0.32%) and (-) - epicatechin (0.23%) (Table S4, Graph S4).

peak	Flavan-3-ols	T_R	% relative value of P long
G	Gallic acid	9.7	0.00
1	B3 (dimer)	34.3	0.00
2	(+)-(+)-catechin	37.6	0.32
3	B1 (dimer)	40.6	0.00
4	T2 (trimer)	45.4	0.00
5	B4 (dimer)	49.9	0.00
6	B2 (dimer)	54.,0	0.00
7	B2-3′-gallate	59.1	0.00
8	(-)-epicatechin	62.9	0.,23
9	B1-3-O-gallate	64.4	0.00
10	C1 (trimer)	67.4	0.00
11	polymers ≥ C_4 units	74.4	99.45
Otros	other flavan-3-ol dimers		

Table S4. Relative distribution of the main flavan-3-ols present in P long by HPLC

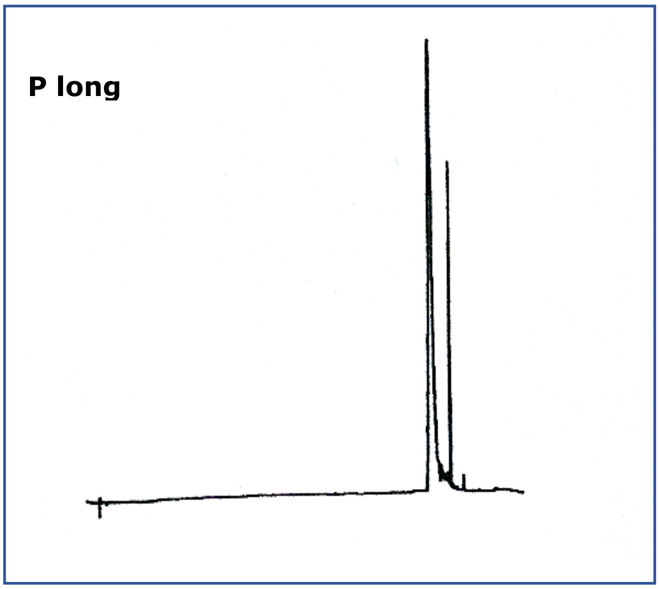

Graph S4. Chromatographic analysis of P long.

5. Soluble Grape Seed Extract

Grape seed extract (Furfural Español S.A., Alcantarilla, Murcia) is a family of the procyanidin compounds: similar to P long and P short but characterized by higher watersolublity. It is made up of a wide variety of active principles, the main ones being catechin and epicatechin. Its general chemical formula is $(C_{15}H_{14}O_6)_n$ (an average of different catechin polymers).It is preparated in a similar way to how P short is prepared. Akin to the rest of the substances evaluated, a solution of the extract was made by adding 0.5 mg of it to a of water.

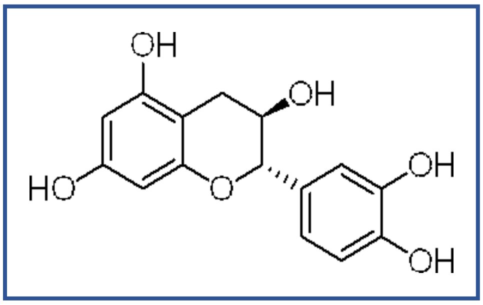

Figure S1. Grape Seed Extract

6. Pomegranate extract

Pomegranate extract (Furfural Español S.A., Alcantarilla, Murcia) consists of powerful antioxidants of which ellagic acid constitutes some 40% and is the most abundant of the polyphenols present. Thus the chemical formula of this extract was considered to be that of its main component ellagic acid ($C_{14}H_6O_8$).

Its was produced in a similar way as the soluble citrus extract but using two different varieties of pomegranate. For its administration to cell culture media, the extract was prepared by dissolving 0.5 mg to one ml of water.

7. Chestnut Wood Extract

Chestnut wood extract (Furfural Español S.A., Alcantarilla, Murcia) is rich in glucosidic tannins and are easily hydrolysed. The extract used in our study contained 17% of the polyphenol known as Castalagin (ellagic acid polymer made up of 5 conjugated ellagic acid molecules and with 5 free trihydroxy groups). This extract is represented by the chemical formula $C_{41}H_{24}O_{26}$.Its was obtained in a similar way to how olive leaf extract was obtained but using chestnut wood powder. For its administration to cell culture media, a solution of the extract was made by adding 0.5 mg of one ml of water.

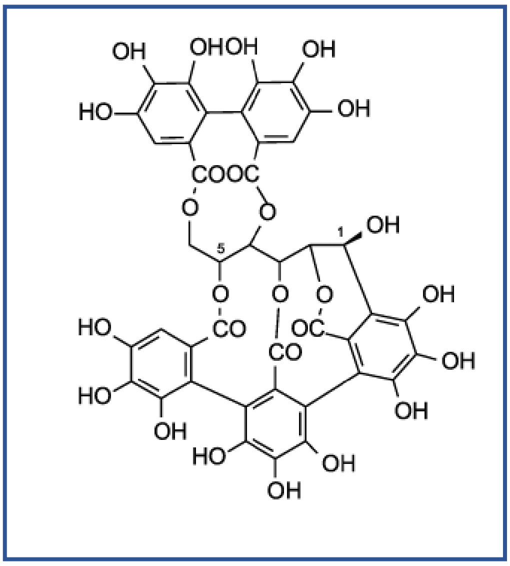

Figure S2. Castalagin (major active component of Chestnut Wood Extract.)

8. Green Tea Extract

Green tea extract (Furfural Español S.A., Alcantarilla, Murcia) contains a class of polyphenols called catechins, mainly epigallocatechin gallate, epicatechin gallate and gallocatechin gallate, known to have various physiological and pharmacological properties.

The extract used in our study was composed of 80% gallocatechins, in which the majority of the active substance was epigallocatechin-3-O-gallate. In addition, the extract was also contained 0.3% caffeine among other substances. The chemical formula of this extract is represented by $C_{22}H_{18}O_{11}$.Its was produced in a similar way as olive leaf extract but using three different varieties of green tea. For its administration to cell culture media, 0.5 mg of the extract was dissolved in water to obtain a concentration of 0.5g/ml.

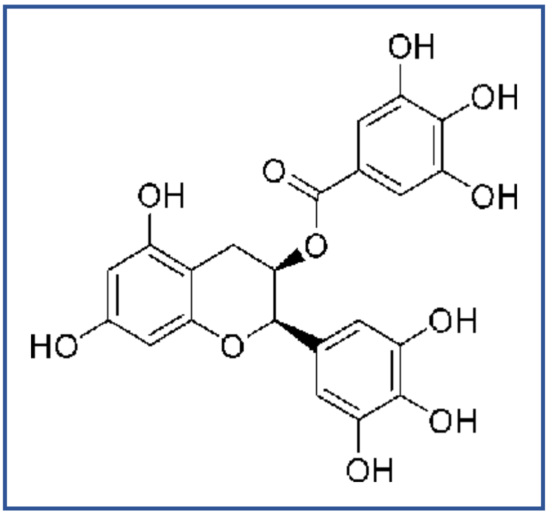

Figure S3. Green Tea Extract

9. Hydroxytyrosol.

Hydroxytyrosol or 3,4-dihydroxyphenylethanol (Nutrafur S.A., Alcantarilla, Murcia) is the main phenolic compound in olive oil either in free or esterified form. It amphipathic with a capacity of capturing free radicals. It defines the shelf-life of the oil by effectively preventing autoxidation. Its chemical formula is $C_8H_{10}O_3$.Hydroxytyrosol used in this work was obtained from olive leaves dried for 48 in an oven at 40 ºC hours. The dried plant material was pulverised and extracted at room temperature in a ethanol: water solvent. Subsequently, the extract was filtered through a cellulose-silica bed using activated carbon as a filter aid to eliminate chlorophyll residues. The resulting solution was concentrated to dryness in vacuo on a rotary evaporator at 60 ° C, producing a yellowish-brown crystalline solid. This solid was suspended in water at a concentration of 20% w/v and the suspension was acidified with 2N hydrochloric acid. This was then heated to 80 ° C for one hour and subsequently cooled to room temperature and filtered to remove all insoluble material. The resulting solution was concentrated on a rotary evaporator at a temperature of 60°C to dryness, obtaining a gummy brown solid, which was recrystallized from methanol to obtain a yellowish crystalline powder. Different evaluations were conducted on this substance in which the concentration used were modified depending on the part of the plant from which it was obtained and the solution that we subsequently prepared before adding the substance to the culture medium. Thus, the following tests were carried out:

1) Hydroxytyrosol-1 was obtained by extracting the fruit of the plant. It was found to contain about 20.3% tyrosol however, there were a small amount of other polyphenols and flavonoids present. It was prepared in a by dissolving 38.5 mg of extract in 20 ml of water.

2) Hydroxytyrosol-6 was obtained from resins of the fruit of the plant which was found to contain 39.2% tyrosol and, together with small amounts of other polyphenols and flavonoids. It was prepared by dissolving 25 mg of the extract in 20 ml of water.

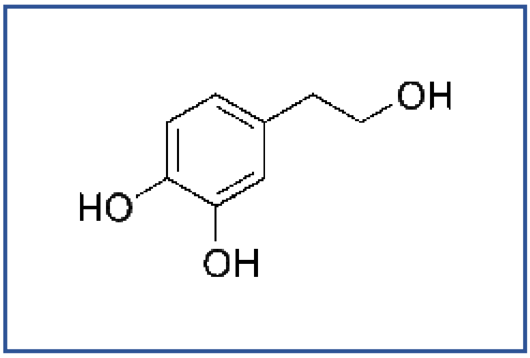

Figure S4. Hydroxytyrosol.
